# Recommendations for Designing, Conducting, and Reporting Feeding Trials in Nutrition Research

**DOI:** 10.1016/j.advnut.2024.100283

**Published:** 2024-08-10

**Authors:** Delyse SY Tien, Meghan Hockey, Daniel So, Jordan Stanford, Erin D Clarke, Clare E Collins, Heidi M Staudacher

**Affiliations:** 1 Food & Mood Centre, Institute for Mental and Physical Health and Clinical Translation (IMPACT), Deakin University, Geelong, Victoria, Australia; 2 Department of Gastroenterology, Monash University and Alfred Health, Melbourne, Victoria, Australia; 3 School of Health Sciences, College of Health Medicine and Wellbeing, the University of Newcastle, New South Wales, Australia; 4 Food and Nutrition Research Program, Hunter Medical Research Institute, New Lambton Heights, New South Wales, Australia

**Keywords:** diet interventions, feeding trials, recommendations, design, conduct, reporting

## Abstract

Double-blind, placebo-controlled, randomized controlled trials are the gold standard for clinical trials in nutrition science. For trials of whole diets, dietary counseling is advantageous as they offer clinical translatability although can vary in the fidelity of the intended intervention from participant to participant and across studies. Feeding trials, in which most or all food is provided, offer high precision and can provide proof-of-concept evidence that a dietary intervention is efficacious and can also better evaluate the effect of known quantities of foods and nutrients on physiology. However, they come with additional methodological complexities. Feeding trials also call for a variety of unique methodological considerations, not least of which relate to the design and delivery of diets to participants. This review aims to provide a comprehensive summary of recommendations for design and conduct of feeding trials, encompassing domiciled and nondomiciled feeding trials. Several pertinent aspects of trial design and methodology are discussed, including defining the study population to maximize retention, safety, and generalizability of findings, recommendations for design of control interventions and optimizing blinding, and specific considerations for clinical populations. A detailed stepwise process for menu design, development, validation, and delivery are also presented. These recommendations aim to facilitate methodologic consistency and execution of high-quality feeding trials, ultimately facilitating improved understanding of the role of diet in treating disease and the underpinning mechanisms.

**Statement of Significance**

Quality design and execution of feeding trials is important to accurately fill the knowledge gaps that cannot be answered by dietary counseling trials alone, so that together, they can help inform evidence-based nutrition practice. However, existing published guidelines either lack comprehensiveness or are dated; therefore, an up-to-date review is much needed for facilitating optimal design, methodology, and reporting of feeding trials.

## Introduction

Rigorous nutrition research is essential to inform national dietary guidelines for health and clinical guidelines for the management of disease and disease risk factors. Clinical trials in nutrition encompass supplementation interventions, in which a single nutrient, food component, or food is added to a pre-existing diet, or whole diet interventions in which several dietary components are concurrently altered [for example, Dietary Approaches to Stop Hypertension diet (DASH diet); fermentable oligosaccharides, disaccharides, monosaccharides and polyols diet (low FODMAP diet); low emulsifier diet]. Whole diets can be delivered to participants via dietary counseling, via providing all or most food (that is, feeding trials) (Table 1), or a hybrid of both [1]. Whole diet counseling trials aim to support participants to change their dietary intake and behaviors through the provision of dietary advice that may or may not be personalized, can be delivered in individual or group settings, and is often supported with provided written information. Although highly clinically translatable, whole diet counseling trials vary in the fidelity of the intended intervention from participant to participant and across studies. Additionally, participant adherence can be influenced by the qualifications and expertise of the researcher providing the advice, the degree of therapeutic alliance, and the frequency of researcher–participant interaction. Furthermore, there is substantial complexity in designing placebo diet interventions to serve as controls for dietary counseling trials, as “inertness” and successful blinding is extremely difficult to achieve [2].

**TABLE 1:** Key differences between whole diet counseling trials and whole diet feeding trials.

	Whole diet counseling trials	Whole diet feeding trials
Setting	Free-living	Fully domiciled, partial-domiciled, or free-living
Duration	Longer duration is possible (months to years)	Typically, short duration (days to months)
Placebo	Challenging to design and implement	Possible to design and implement
Blinding	Impossible to double-blind, possible to single-blind	Possible to double-blind
Adherence	Variable between participants and may depend on factors such as researcher–participant rapport	High adherence possible
Cost	Low cost	Costly
Resources	Clinician experienced in dietary counseling may be required	Blinded and unblinded staff required Logistically demanding
Application of findings	Real-world effectiveness and clinical translatability	Proof-of-concept for therapeutic benefit Advances understanding of physiological mechanisms

Feeding trials involve the provision of food to participants, and in some cases, beverages, over the course of the intervention period. Complete provision of foods is ideal and preferred in achieving proof-of-concept evidence that a dietary intervention is efficacious [3] and for evaluating the effect of known quantities of foods and nutrients on physiology; however, some feeding studies only provided foods partially [4]. Although feeding trials are costly and resource intensive with lower external validity compared with dietary counseling trials, the provision of foods offers high intervention accuracy through maximizing adherence (partly because of the reduced burden of participant meal preparation) and enables design of blinded placebo treatments. Feeding trials can also facilitate the identification of objective biomarkers of adherence to highly defined dietary exposures [4]. This is of extreme appeal given the rising interest in personalized nutrition coupled with improved analytical capabilities of human biological samples.

Feeding trials can be conducted in fully domiciled, partial-domiciled, or nondomiciled settings (Table 2 [5–10]). Fully domiciled feeding trials involve participants living in a tightly controlled environment such as in a metabolic chamber, inpatient research facility, or institutional setting. This approach offers extreme control over the delivery of the research diet and direct measurement of dietary intake, control over environmental influences, and facilitates objective biomarker monitoring in real time. However, such precision comes with high financial cost, high participant burden, and requires major resources. Partial-domiciled feeding trials present an alternative in situations where tight control of environmental factors and real-time monitoring is not needed, or where there are financial and/or resource constraints. In these trials, participants consume some or all meals on-site and are then allowed to return home. Finally, in nondomiciled feeding trials, meals are provided to participants to be consumed at home. These are the most common type of feeding trial and are more susceptible to lower adherence compared with fully domiciled trials, but are less costly, more practical for participants, and offer greater precision compared with counseling trials.

**TABLE 2:** Types of feeding trials.

Type	Setting	Example
Fully domiciled	Participants reside at the research facility (for example, metabolic chamber, inpatient research facility)	Effect of restricted carbohydrate vs. restricted fat on bodyweight [5] Effect of diet high in ultraprocessed foods on energy intake [6]

Partial-domiciled	Participants consume some or all meals at the research facility and are able to spend a majority of the intervention at home	Effect of time-restricted eating on weight change [7] Effect of Chinese Heart-Healthy Diet on blood pressure [8]

Nondomiciled	Meals are provided to participants for consumption at home	Effect of DASH diet on blood pressure [9] Effect of Dietary Guidelines for Americans-based diet on cardiometabolic health indices [10]

Abbreviation: DASH, Dietary Approaches to Stop Hypertension.

Feeding trials offer several clear advantages over dietary counseling trials, but are complex to design and implement. Quality design and execution of feeding trials is needed to fill the knowledge gaps that cannot be answered by dietary counseling trials alone, so that together, they can help inform evidence-based nutrition practice. Published guidelines exist for the design and implementation of feeding trials. However, some are based on experiences bespoke to specific trials [11,12] and, therefore, miss certain critical facets, such as those relevant to domiciled feeding trials. Others review the design and conduct of dietary trials, but with only minimal recommendations dedicated to those designed as feeding trials [1,3,13]. Many of the more comprehensive publications guiding feeding trial methodology are now over 10 years old [14,15]. Therefore, we aim to review the relevant literature and provide recommendations for the design, methodology, and reporting of feeding trials (Table 3). We also provide specific recommendations for the design of menus for feeding trials and specific factors to consider when evaluating clinical populations.

**TABLE 3:** Recommendations for feeding trial design, methodology, and reporting.

Research team	- Involve a dietitian/qualified clinical nutrition researcher early in the planning stage and throughout. Diet expertise is critical for design and conduct of the research, dietary assessment, analysis, and interpretation - Medical specialists should be an integral part of the research team when studying clinical populations

Trial design	- Consider the advantages and disadvantages of crossover designs, particularly the duration of washout - Provide all or most food with minimal preparation where possible to optimize adherence. Justification should be made for decisions on how much food is provided

Trial duration	- Implement trial for minimum duration of 2–4 wk

Sample size	- Clearly report sample size calculation and data used in calculation - Accept calculation may be an overestimate if data used to calculate sample size is from counseling trials

Inclusion and exclusion criteria	- Generally, these should be stringent enough to ensure that the research question can be answered and adherence optimized, however general enough to maintain some generalizability - Recommend exclude: eating disorders, “not able to consume diet provided”, food allergy, severe food intolerance

Informed consent process	- Consider limited disclosure of research aims within ethical boundaries for blinding purposes - Consider showing sample menu to individuals to help them decide whether to participate - Clearly state whether all, most, or some food is provided - Clearly state restrictions associated with participation, for example, travel

Intervention and control design	- Describe and report the diet targets, rationale for diet targets, foods provided, consider providing meal plans in supplementary information - Describe and report if control diet is an active comparator or designed to be placebo - Consider and report attempts made to fit menu within target population’s energy requirements

Blinding	- Double-blinding is recommended and if single-blinding is used, the reason for this should be transparently reported - Develop a plan and report on who is blinded, how blinding will be maintained, and when and how to evaluate blinding success

Dietary assessment	- Use precise diet assessment methods (for example, observation for fully domiciled or weighed food records for nondomiciled) - Use food and drink checklists for recording food consumed to minimize participant burden

Adherence	- Establish and report adherence target and how adherence will be measured *a priori* - Offer “free foods” for participants with higher energy needs - Where possible, use objective dietary biomarkers (for example, plasma carotenoids)

Bias	- Attempt to double-blind where possible to minimize bias - Acknowledge selection bias may be more likely because of stringent inclusion and exclusion criteria and the requirements of feeding trials

Tolerability and acceptability of diets	- Measure tolerability and acceptability to assess real-world applicability

Ethical considerations	- Consider the duration of study and nutritional adequacy of diets - Incorporate participant physical safeguards (for example, physician approval for involvement of high-risk patients, regular bodyweight monitoring) and mental safeguards (for example, regular mental health monitoring)

Reporting	- Use available reporting guidelines for dietary trials and development of control interventions

### Trial design

There are 2 major considerations in planning the design of a feeding trial. Firstly, there is the choice of whether it is designed as a parallel-design or crossover trial. Feeding trials are commonly designed as crossover trials, in which participants are allocated to each treatment in random order. Crossover designs have notable advantage over parallel designs, reducing intra-individual variability thereby requiring fewer participants. However, the duration of participation is extended as participant is required across all treatments. A specific drawback of a crossover trial design is the carryover of effects between treatments [16]. While implementation of a washout period between treatment phases may mitigate such carryover, the optimal duration of washout, which would vary on the basis of the proposed mechanism of action and the duration of the effect of the dietary component(s) being investigated, is often not known. Statistical analysis of carryover effects also has limited power and risk of type II error (that is, incorrect assertion that carryover is not present) [17].

Secondly, for feeding studies that are not fully domiciled, researchers must consider whether all or some food is provided to participants. Often all food is provided to participants, although some studies provide a majority of food, or key foods, and provide advice to participants for sourcing and preparing the remainder of food items for the duration of the study. Where possible, it is recommended that all food is provided. Best adherence is undoubtedly achieved with the provision of all food to participants, although it is more costly and requires substantially more operational planning than only providing key foods.

### Study duration

Typically, feeding trials are shorter in duration than dietary counseling trials, and this may be a reflection of their proof-of-concept nature and not effectiveness in the “real world.” In a recent synthesis of feeding trials designed to quantify the diet-related metabolome, 19 of 50 studies were of ≤1–4 wk duration [4]. Factors influencing the study duration include the time required for a diet to elicit the biological response of interest and the study resources available. Broadly speaking, a minimum duration of 2–4 wk is recommended to allow sufficient time for diet to elicit a biological response. However, it is important to note that biological responses can vary greatly, from extremely rapidly to years before detectable. For example, few feeding trials have observed post-prandial fluctuations in gut hormones and serum short chain fatty acids within hours [18], whereas gut microbiome changes were seen within a day in response to diet alterations [19]. For studies involving dietary or physiologic outcomes that may be directly influenced by endogenous sex hormonal alterations across the menstrual cycle [20] (for example, ad libitum dietary intake [21] or gastrointestinal symptoms [22]), 4 wk should be considered, while acknowledging that not all responses are affected by menstrual cycle.

### Sample size

Feeding trials generally include small participant numbers (a majority include *n* < 50) [4], although a few larger whole diet feeding trials have been conducted, such as those evaluating the effect of diet on hypertension (for example, DASH diet feeding trial (*n* = 459) [9] and the Diet, Exercise and Cardiovascular health-Diet trial (*n* = 265) [8]). The smaller sample sizes can often be attributed to the nature of feeding trials, which are conducted to provide proof-of-concept evidence of efficacy. Some proof-of-concept trials calculate a sample size informed by relevant previously published data [6,23], others use either very conservative calculations or do not calculate one at all. It is worth noting that estimating effect sizes and the spread of effects for feeding trials may require extraction of data from trials that do not involve provision of food and in which treatment outcomes will likely be more variable, and this may ultimately lead to the overestimation of sample size. In these cases, this should be stated and other methods of determining samples sizes should be used (for example, convenience sample, available resources) [16].

### Inclusion and exclusion criteria

As for any other clinical trial, participants in a feeding trial should be selected on the basis of features necessary to answer the research question, including demographic, clinical and geographic characteristics [3]. However, there are unique considerations when developing exclusion criteria, as personalization of diet is much more difficult than that for diet counseling trials. These considerations may include factors such as dietary restrictions (for example, vegetarianism or veganism), presence of food allergies or intolerances or other clinical conditions, specific cultural or religious dietary patterns, or recent adherence to a special diet, that all may influence participants’ ability to fully adhere to the diets provided. People engaging in excessive physical activity may not be suitable if for the purposes of the trial participants must remain bodyweight stable. A general exclusion criterion such as “participants who are unable to consume the diet provided” could be considered. Presenting potential participants with a sample meal plan or offering tastings of typical menu items during screening may help adequately answer this question [3]. This may be particularly useful in studies in which effects of an extreme diet (for example, very low calorie diet) are assessed. It is recommended that individuals with eating disorders or disordered eating be excluded from feeding trials, a criterion that many feeding studies have introduced [[24], [25], [26], [27]] as strict regimens outside of eating disorder specialist care are generally considered unsafe in this group. Validated questionnaires such as the Sick, Control, One, Fat, Food [28] or Eating Disorder Examination Questionnaire [29] may be used to screen for disordered eating behaviors. Nondomiciled feeding trials should consider whether access to kitchen facilities or minimum cooking skill requirements determines eligibility as reheating and/or assembling of meals is often necessary. Alternatively, additional resources may be provided to individuals without usual access to these facilities to circumvent excluding these participants.

### Informed consent process

The informed consent process in feeding trials, as with any other clinical trial, should comply with applicable regulatory requirements and adhere to Good Clinical Practice. To facilitate informed decision making, the plain language statement should clearly state whether participants are provided with some, most, or all food as part of the trial, and the degree of preparation required. Restrictions associated with trial participation (for example, dining out, travel), and for blinded studies, the reason for concealing the diet(s) should also be detailed (see later section on blinding and controls). As described above, the use of a sample menu, inclusive of portion sizes, is recommended during the informed consent process to ensure that participants are fully aware of what is expected of them during the trial.

### Blinding and controls

Blinding is a critical aspect of placebo-controlled trials that aims to minimize the effects of expectation bias. Where feasible, key members of the research team should be blinded, including participants, data collectors, outcome assessors, and staff undertaking laboratory analysis of biological samples. A major advantage of feeding trials compared with dietary counseling trials is the greater ability to blind participants to treatment allocation. However, it should be acknowledged that blinding, and particularly double-blinding, of whole diets is challenging and requires careful planning and execution.

Placebo controls are more feasible in feeding trials than in counseling trials as diets can be carefully designed to be “inert” while matched for all other dietary components not under investigation [2]. Appearance can also be matched as closely as possible and storage in opaque containers can additionally safeguard blinding [15]. Placebo diets should ideally represent the study populations’ “usual” diet and are often based on relevant national nutrition survey data [10,24] or national apparent consumption data [30]. For clinical populations, it is important to note that “usual diets” may be very different to the national average diet, and this could be factored into placebo diet design.

Alternatively, feeding trials may incorporate an active control intervention, rather than placebo. This may occur when the aim is to test whether an intervention diet has equal or greater efficacy than a diet reflective of standard practice. For example, several trials have investigated the effects of varying macronutrient compositions on cardiometabolic outcomes [[31], [32], [33], [34]], and the effects of a specific carbohydrate diet have been compared with a Mediterranean diet to manage symptoms of Crohn’s disease [35]. Double-blinding of treatment allocation is also feasible in active comparator feeding trials.

Additional measures need to be considered to incorporate and maintain double-blinding in feeding trials. These include consideration of how to manage inevitable participant diet queries (for example, questions regarding meal preparation) and mention of the diet by participants during study visits with the blinded researcher. The access to study information that might risk unblinding should be restricted to the blinded researcher (for example, online meal ordering platforms). Contact between participants and blinded researchers during the intervention phase should be limited as much as possible. Double-blinding essentially requires a second team of staff to conduct trial tasks and, therefore, comes at additional expense.

Participants may also be blinded to the intent of the trial. For example, if participants are recruited to a trial in which 2 very different dietary patterns are compared, concealing the intent of the study may minimize expectancy bias even if participants correctly guess their dietary treatment. If the intent of the trial is blinded, then care must be taken to ensure strict concealment of the intent in ethics-approved recruitment materials and participant-facing materials.

Blinding success is rarely reported in dietary trials; however, it is increasingly recommended in clinical trials generally and has been included in the recent control intervention reporting guidelines [36,37]. To evaluate the success of blinding, participants can be asked to guess their treatment assignments on a binary scale (intervention or placebo), or a 3-point (intervention, placebo, or do not know) or 5-point scale (strongly believe the treatment is intervention, somewhat believe the treatment is intervention, do not know, somewhat believe the treatment is placebo, or strongly believe the treatment is placebo) [38]. The success of blinding should be reported in the final manuscript using contingency tables or can be evaluated statistically using Cohen’s kappa or a chi-square test [39,40], or validated blinding indices (BI) such as the James’ BI [41] and Bang’s BI [42].

Overall, it is recommended that feeding trials are designed to be double-blind where possible (that is, participants and researchers conducting trial visits are blinded) and that outcome assessors are also blinded to allocation. A comprehensive plan should be developed early in the design phase of the trial that sets out who will be blinded, how blinding will be maintained, and when and how to evaluate blinding success. If a single-blind approach is chosen, then the reason for this should be provided in the reporting of findings.

### Run-in periods

Run-in periods are phases that occur after participant inclusion but before randomization and help refine the selection of patients for the randomization phase of the trial. These may be particularly relevant for proof-of-concept trials in which a treatment will be tested in ideal conditions [43]. A run-in can be used to standardize the participant population, thereby increasing the statistical power to detect treatment effects. For example, run-ins have been used to homogenize baseline diet before commencing the treatment period [26,44]. Alternatively, run-in periods can also help identify individuals who are not able to adhere to the diet before randomization [45], again enhancing the chance of detecting treatment effects. Run-ins may also be useful to estimate energy needs of participants or to allow adaptation to the intervention diet so as to reduce risk of side effects in the main trial (for example, gradual increase in fiber intake in a high-fiber intervention trial [46]).

Although run-in periods offer advantages, they increase the total duration of a trial, and therefore cost, and impact external validity of the trial. Additionally, in the case of run-in periods that require participants to alter their diet, this may inadvertently lead to an improvement in symptoms or health status of the participants, potentially counteracting the potential benefits of the test diet [3].

### Dietary assessment

Dietary assessment is essential in feeding trials to measure adherence and for measuring intervention composition and nutritional adequacy. A large variety of traditional assessment tools are available, including observation, daily food diaries or checklists, weighed and unweighed food records, and 24-h diet recalls. Many of these tools have been reviewed in detail elsewhere [47]. Bodyweight monitoring may also help support adherence monitoring, particularly where energy provision is matched to individual participant energy needs [8,48].

Dietary assessment via observation of all eating occasions is possible in domiciled feeding trials, and together with measured weights of provided and uneaten food, can facilitate very accurate estimates of dietary intake [15]. For trials that are not fully domiciled, weighed food records, in which participants weigh and record all consumed food eaten external to the research center, are considered the most precise measure of dietary intake [49] and should be used where feasible. Alternatively, participants can be asked to freeze all leftover uneaten food, which is returned to researchers then subsequently weighed, and the difference in nutrition composition between provided and leftover food can be calculated. Where possible, the separation of mixed meals into components, although tedious, should be attempted and each component weighed. Image-based diet capture of remaining foods or meals may also improve the accuracy of conventional diet assessment methods [50].

Participants should be provided with clear instructions as to how to record their dietary data to enhance data accuracy. Data should be thoroughly cross-checked with the participant as close as possible to completion time, especially considering over 90% of participant diet records are incorrect before dietitian verification [51]. Prolonged diet recording periods should be avoided to minimize recording fatigue and response bias. The use of food and drink checklists can minimize participant burden. Baseline diet assessment before allocation should occur via traditional diet assessment methods because checklists will not fully assess participants’ habitual diet. Any food or beverages outside the trial diet, although discouraged, should be clearly recorded.

Emerging technologies, including image-assisted and image-based dietary assessment methods, as mentioned above, will further reduce respondent burden and improve accuracy of reporting [50]. However, these methods are not considered useful as a standalone assessment [52]. In some cases, validated dietary biomarkers as objective adherence measures could be implemented, such as skin and plasma carotenoid responses assessed by spectroscopy for measuring adherence to a fruit and vegetable-rich diet [53] in addition to the use of traditional diet assessment methods. The use of validated dietary biomarkers as objective markers of adherence is becoming increasingly important as analytical capabilities improve and novel biomarkers are identified [54].

### Adherence criteria

Dietary adherence in feeding trials refers to the degree to which participants consume the foods and fluids provided to them during the period of the intervention. Deviations from the intended intervention may occur for a range of reasons, such as dislike of intervention menu items, specific food preferences, insufficient food to meet caloric needs, attendance at special occasions, and sweet/salty cravings [12,55,56]. Deviations in nonfully domiciled feeding trials can range from being benign and having only a minor impact on participant adherence (for example, consumption of low-calorie foods with minimal nutritional value) or can be major and lead to major impact on adherence (for example, regular consumption of foods not allowed on the intervention diet). The threshold for adherence in a given trial should be defined *a priori*. This is usually a threshold proportion of food provided (often 80%) [57,58]. Additional criteria relating to the consumption of external foods or key foods relevant to the intervention may also be specified.

### Bias

A major advantage of feeding trials over dietary counseling trials is the reduced exposure to several types of bias [1]. Expectation bias can be minimized in feeding trials as participants can be blinded to their allocation, and even to the intent of the study. Confirmation bias, in which the investigator favors 1 treatment over another on the basis of their existing beliefs or biases, can also be completely avoided if the researcher is blinded to the intervention allocation. Additionally, most feeding trials avoid the need for dietary counseling, and so even single-blind trials may elicit minimal confirmation bias because of the reduced need for interaction between the researcher and participant. To the contrary, selection bias may be enhanced in feeding trials. Specific groups of individuals may volunteer for research because of the appeal of free food and may be more available or willing to commit to fully and partial-domiciled feeding trials. This may lead to a study population that is less representative than intended.

### Measuring tolerability and acceptability

Feeding trials in which the primary aim is to assess the therapeutic effect of a diet (that is, rather than assessing mechanisms) should consider measuring participant tolerability and acceptability of the intervention. Specifically, tolerability refers to the occurrence of gastrointestinal or other physical symptoms in response to diet, impact of the diet on satiety and hunger, and participant ratings of food volume [1]. These can be assessed via instruments or scales validated in the population of interest, and subjective reporting of the adequacy of food volume. Acceptability of the trial diet in terms of taste can also be measured using existing, although largely unvalidated, tools (for example, Diet Satisfaction Questionnaire [59], Diet Acceptability Questionnaire [12]) or user-developed tools for the specific study.

### Ethical and safety considerations

There are a range of ethical and safety concerns that must be considered in feeding trials. Narrow eligibility criteria may require in depth justification to address local institutional review board queries about recruitment from a diverse population. Where limited disclosure of the intent of the trial is required, this will also require careful justification. Domiciled feeding studies may attract a range of ethical concerns relating to the physical and mental health of participants who are required to reside outside their own home for considerable periods of time for research purposes. For extreme interventions, physician approval should be considered for individuals at high risk of health compromise as a result of participation. For the safety of participants, regular monitoring of participants, including any reported side effects from the dietary interventions, is recommended with escalation care plans in place, particularly for vulnerable populations or when extreme diets are being investigated.

Body weight should be monitored regularly throughout in all feeding trials. Provision of scales for participants should be considered for nondomiciled trials. Where weight loss is not a research outcome, a weight loss threshold should be set that signals the need for adjustment in quantities of food provided. For example, in a trial evaluating the effect of the DASH diet on blood pressure, bodyweight of participants was measured frequently and energy provision adjusted to ensure bodyweight remained within ±2% of baseline bodyweight [60]. Comprehensive assessment of baseline habitual diet of participants will assist finetuning calculation of energy requirements. Specific dietary adjustments may be needed in cases of bodyweight loss and/or hunger because of insufficient food, and appropriate actions in these cases should be planned and documented in standard operating procedures (SOPs). For example, a simple strategy of adding “unit” foods, typically nutrient-dense foods providing a total of 100 kcal, can be implemented [7,12,61]. Clinical monitoring of blood pressure, blood lipids, glucose, and/or liver function may also be necessary. Ensuring continuity of care should also be considered for clinical populations. This may include feedback about the patients’ trial participation and outcomes to the individual’s healthcare team or onward referral for ongoing dietetic support.

Food safety is an additional critical consideration in feeding trials. Food safety practices must be employed and relevant records of training and monitoring (for example, fridge temperatures) must be in place for on-site food preparation and for commercial food service providers. Prepared meals should be produced in accordance with current Good Manufacturing Practices and microbiological testing may be required. SOPs for cooking and storage should be in place and all processes and/or deviations need to be well-documented [62]. A research agreement between the research institution and commercial food service companies can be used to detail relevant food safety information including the use of meals within expiry timeframe.

### Reporting

A recent scoping review of human feeding study methodologies identified the need for more detailed reporting of feeding trial design and methods [4]. All reporting should follow appropriate reporting guidelines, such as the Consolidated Standards of Reporting Trials (CONSORT) for randomized controlled trials. The development of a CONSORT-nutrition-specific extension is currently underway and will enhance high-quality reporting of dietary clinical trials [63]. Resources such as the Enhancing the Quality And Transparency of health Research Network are recommended to identify the appropriate reporting guidelines. Reporting guidelines relevant to dietary trials [1] and the Control interventions in efficacy and mechanistic trials of Physical, Psychological and Self-management therapies (CoPPS) statement [36] should be considered.

In addition to the above, for feeding trials, the following should be reported at a minimum: granular reporting of the provided diets, including descriptions of food(s) and beverages, proportion of total food provided, example meal plans; the level of blinding implemented and how it was maintained with reference to presentation and appearance of the meals and all other attempts to maintain participant and researcher blinding; rationales for the choice of control diet used; adherence threshold used and measured adherence to the provided diets.

### Preliminary planning of trial interventions

A variety of decisions need to be made early in the planning phase to guide subsequent menu design, development, and execution. An experienced dietitian or experienced clinical nutritionist is essential from the preliminary planning phase through to trial implementation and final analysis and interpretation. Once the nature of the intervention and control diets has been decided, the research team should select the mode of diet preparation and delivery. This may include 1 or a hybrid of options such as: fresh food delivered to/picked up and prepared at home by participants [64], ready-meals and snacks from commercial supermarkets delivered to/picked up by participants [30], and specifically prepared fresh or frozen meals cooked by the research team or a commercial service delivered to/picked up by participants [12,[65], [66], [67]]. Next, a decision must be made as to whether a menu plan is offered at a single energy level or is offered at multiple energy levels, in which additional items are added to core meals and snacks. For partial or nondomiciled trials, the incorporation of on-site dining, which might improve adherence [61], must also be considered.

For nondomiciled feeding trials, it is recommended that food items provided to participants require minimal food preparation. For trials in which food is provided by supermarket delivery, it is preferable to use fresh products (for example, fruit, vegetables) that are available year-round and generic brands of packaged foods to ensure availability throughout the entire trial period. Additional considerations warranting discussion during trial planning include determining whether participants will be permitted to consume external meals at a predetermined frequency, allowance of specific beverages (for example, alcohol), inclusion of recommended “free foods” for participants who are still hungry after consuming study meals, and provision of meal substitutions for when a participant may not tolerate a designated meal or for unforeseen circumstances.

### Menu design, development, validation, and delivery

**FIGURE 1:**
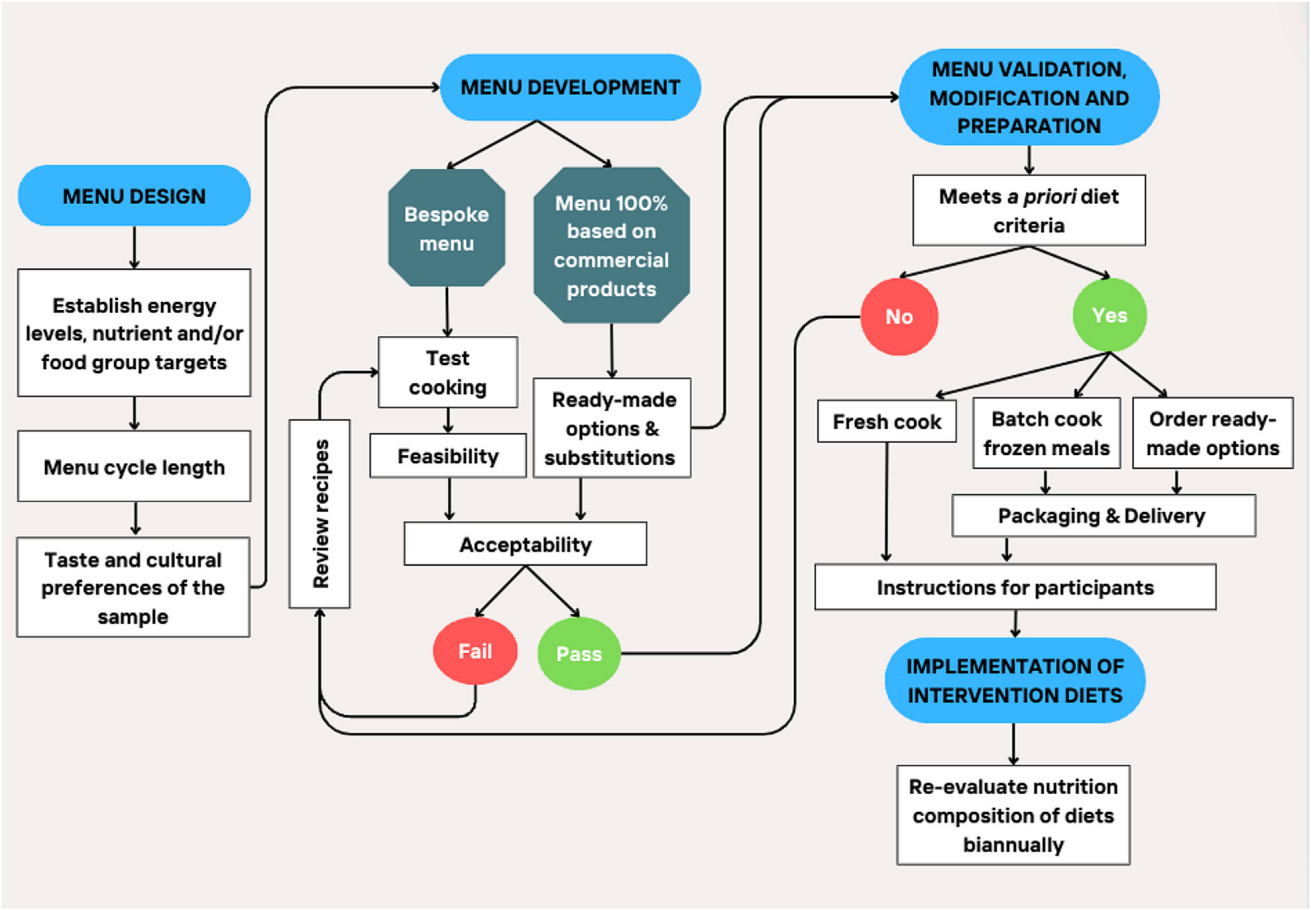
Recommended steps for menu design and development, and implementation of intervention diets in feeding trials. These steps can be applied to a range of whole diet interventions. For feeding trials with menu 100% on the basis of commercial products (that is, supermarket ready-made options), the menu is iteratively validated throughout the development phase with substitutions selected concurrently.

We propose a stepwise approach for the design, development, and delivery of the intervention in feeding trials (Figure 1).

#### Menu design

The first step for designing a menu is establishing diet targets for the intervention and control diets. These targets may be nutrient-specific (for example, high-fiber diet) or food group-specific (for example, diet on the basis of national dietary guidelines). For example, a trial testing the impact of a low-carbohydrate diet on a specific clinical or physiological outcome would require not only carbohydrate targets (for example, absolute or relative carbohydrate content) but also for protein and fat. Where possible, test diets should be nutritionally adequate on the basis of sex and age of the participants. Where several menu plans are to be constructed on the basis of differing energy requirements, these energy levels need to be set (for example, 2000 kcal, 2400 kcal, and 2800 kcal) [7,68,69]. An alternative option, though uncommon, is the provision of a menu plan at 1 energy level to all participants and the provision of instructions for participants to consume a consistent proportion of each meal [70,71]. The second step for menu design is ensuring that the menu cycle is of an appropriate duration to prevent participant menu fatigue. Typically, this will be a 3-d cycle for a short-term feeding trial (<1 wk), or a 7-d cycle for a longer trial duration, which incorporates weekday and weekend menus [15]. The third step, where possible, is to conceptualize meals that meet the taste and cultural preference of the sample to enhance acceptability and adherence.

#### Menu development

After the general structure of the meal plans has been finalized, core meals can be conceptualized and developed. These steps differ depending on whether bespoke cooked meals prepared by the research team or a commercial meal provider, or ready-made supermarket meal options are to be provided. For ready-made supermarket options, suitable substitutions should be selected in cases of lack of availability. For bespoke meals prepared in an on-site kitchen or in collaboration with a food service provider or meal delivery company, recipes must be developed by the research team and should undergo test cooking to determine feasibility of cost, preparation time, and freeze/thaw quality where relevant. Subsequent taste-testing by the research team or volunteers should be undertaken to assess palatability, flavor profile, appearance, texture, and portion size [12,61]. For studies in clinical populations, patient and public involvement can be extremely useful. Where meals do not meet expected standards, research and kitchen staff should work together to modify or replace recipes, conduct further test cooks, and retest acceptability of meals [61]. Researchers should factor in sufficient time for this iterative test cooking process.

#### Menu validation, modification, and preparation

Menu validation involves rigorously analyzing the final menu plan for nutrition composition and comparing it to the predetermined diet targets. This step is relevant only for trials in which meals or components of the menu plan are to be prepared bespoke for the trial, as for trials in which foods are to be delivered by supermarket delivery, the menu should have been iteratively validated throughout the development phase. An *a priori* upper and lower range acceptable validation threshold should be established for specific diet targets (for example, ±10% of target nutrient) [11]. For domiciled trials, the validation process should be conducted using chemical analysis [72] if extreme precision is required and the research budget permits. For most nondomiciled feeding trials, nutrition analysis software that utilizes comprehensive, country-specific food composition databases should be used. Food provided and/or recipes will require modification if the first version of the menu plan fails validation. Collinearity of nutrition variables can challenge this process, particularly where several diet targets have been set [73]. For example, in a feeding trial testing varying content of dietary emulsifiers, reducing foods high in emulsifiers may impact on other nutrients, such as reducing total sugar intake [74]. Minor adjustments to a menu may lead to changes in the contribution of multiple nutrients or foods, and many adjustments may need to be made to ensure that the final menu meets the full set of diet targets. It is recommended that researchers plan sufficient time for this process.

Given the complexities of menu design, development, and validation for feeding trials, the use of a mixed integer linear programming model has recently been explored to streamline the design process and reduce development costs in feeding trials [75]. This model optimizes menu design by efficiently and practically producing menus that adhere to all predetermined criteria on the basis of energy requirements. This model has been shown to design menus with greater reproducibility and objectivity when compared with manual design methods that rely solely on the expertise of the research team; however, it does require a modeler with appropriate expertise.

Once the menu plan is finalized, fresh cooking can be planned and batch cooking can occur for frozen meals. Multiple food preparation sessions may be required throughout the recruitment period for frozen meals depending on shelf life. The quantity of meals to be prepared in each batch should be estimated according to the anticipated recruitment rate to reduce the likelihood of food wastage. All recipes (for example, foods, brand names, raw/cooked weights of ingredients) and food preparation procedures, including cooking temperatures and times and cooking techniques should be recorded and standardized across batches to ensure that the nutrition composition of meals is identical from first to last participant [15].

#### Packaging and delivery

For double-blinded trials using bespoke meals prepared for the trial, packaging and labeling of meals needs careful consideration. Meal labels should provide adequate yet generic description of all foods to assist in maintaining blinding, and should also include details about heating and storage instructions [62]. Participants and researchers must be able to identify the correct meal for each mealtime but remain blinded to allocation. Meals can either be delivered to participants’ homes or collected from the research center. In most cases, meals will be provided in disposable containers that should be sufficiently durable to withstand heating, should be leak-proof, and should be packed appropriately to ensure appropriate temperature during transportation [62]. Supermarket items can be delivered to participants’ homes or collected by participants via “click and collect” which may be a convenient alternative but comes with less researcher control over unavailable items.

#### Participant instructions

Detailed instructions regarding the diet should be provided to participants at the first research visit or at the time of diet collection/delivery. These should include information regarding the importance of consuming all foods provided, reporting losses because of spillage, advice regarding items such as seasonings, specific beverages and “free foods”, the choice and quantity of “free foods” allowed, food preparation instructions or recipes where required, information about handling and storage of the food items/meals, and the use of emergency meals where appropriate [7,12,61,62]. Detailed instructions about recording dietary intake should also be provided or reiterated at this time. Researcher contact details should be provided in case of participant queries.

### Implementing feeding interventions in clinical populations

Although many feeding trials are conducted in healthy individuals, individuals with a disease or disease risk factors are also often recruited to feeding trials. Particularly in extreme diet intervention trials, these groups may be particularly vulnerable to changes in bodyweight and/or metabolism. Medical specialists should be considered as a key part of the trial team and may undertake medical clearance assessments and provide ongoing monitoring for participant safety. A robust safety monitoring plan should be put in place and all researchers who have patient contact should be familiar with how to escalate care where needed. Diets provided to individuals with gastrointestinal disorders, and/or to those who habitually experience gastrointestinal symptoms or may be sensitive to dietary change, may need to be designed in more granular detail beyond macro- and micronutrients. For example, the content of FODMAPs or gluten may need to be considered, to ensure tolerability. For individuals with mental health symptoms, careful screening for eating disorders and disordered eating must be implemented. Substance misuse may be common in populations with mental health conditions and may influence the ability of participants to consume all provided food. Low-burden diet assessment tools, clear instructions for food preparation, and additional dietetic time may be needed for people with mental health concerns, as symptoms of apathy, impaired cognitive processes, and poor concentration are frequently reported.

In conclusion, feeding trials provide nutrition science evidence that is not achievable with epidemiological research or standard randomized controlled trials on the basis of dietary counseling. Highly precise dietary interventions can be designed that are able to be double-blinded and placebo-controlled, and are capable of eliciting proof-of-concept causal evidence for the role of diet in disease, advancing mechanistic understanding and facilitating biomarker identification for adherence to highly defined diets. However, to add to our current understanding, they must be on the basis of multidisciplinary collaboration, careful design and conduct, and transparent trial reporting. The recommendations detailed here aim to improve the quality of future feeding trials and ultimately enhance nutrition discovery.

## Author contributions

The authors’ responsibilities were as follows – DSYT, HMS: conceptualized the overall design of the manuscript; all authors had responsibility for writing the manuscript; DSYT, HMS: finalized the content and revised the manuscript; and all authors: read and approved the final manuscript.

## Conflict of interest

HMS has received research funding from DSM Pharmaceuticals, the Rome Foundation, and consulting fees from Dietitian Connection, Dietitians Australia and Microba. These companies have no input into the manuscript. All other authors report no conflicts of interest.

## Funding

HMS is currently supported by a National Health and Medical Research Council emerging leadership fellowship (APP2018118). CEC is supported by a NHMRC leadership fellowship (APP2009340). DSYT is supported by a Deakin University Postgraduate Research Scholarship.
